# Physical and Mental Aspects of Quality of Life in Patients With Charcot-Marie-Tooth Disease Type 1A

**DOI:** 10.3389/fneur.2022.852150

**Published:** 2022-03-16

**Authors:** Vukan Ivanovic, Bogdan Bjelica, Aleksa Palibrk, Marija Brankovic, Ivo Bozovic, Ivana Basta, Andrija Savic, Vidosava Rakocevic Stojanovic, Aleksandra Kacar

**Affiliations:** ^1^Department of Neurology Clinic, Faculty of Medicine, University Clinical Center of Serbia, University of Belgrade, Belgrade, Serbia; ^2^Department of Neurology, Hanover Medical School, Hanover, Germany; ^3^Department of Neurosurgery Clinic, Faculty of Medicine, University Clinical Center of Serbia, University of Belgrade, Belgrade, Serbia

**Keywords:** Charcot-Marie-Tooth type 1A (CMT1A), quality of life, impairment, disability, fatigue, depression

## Abstract

**Introduction:**

Charcot-Marie-Tooth type 1A (CMT1A) comprises ~50% of all CMT cases. CMT1A is a slowly progressive motor and sensory neuropathy that leads to significant disability. We aimed to investigate the quality of life (QoL) in Serbian patients with CMT1A and to assess sociodemographic and clinical features associated with their QoL.

**Material and Methods:**

Forty-five genetically confirmed patients with CMT1A were included −60% women [age 50.4 ± 12.6 years, disease duration 22 (12.5–31.5) years]. SF-36, Medical Research Council (MRC) Sum Score, CMT Examination Score (CMTES), Overall Neuropathy Limitation Scale (ONLS), Beck Depression Inventory (BDI), and Krupp's Fatigue Severity Scale (FSS) were used in the study.

**Results:**

Regarding SF-36, Mental Health and Social Functioning were the scales with the best achievements, whereas Role Physical was the worst domain. Worse QoL in patients with CMT1A was associated with elder age (rho = −0.34, *p* < 0.05), longer disease duration (rho = −0.31, *p* < 0.05), more pronounced muscle weakness measured by MRC-SS (rho = 0.43, *p* < 0.01), presence of tremor (*p* < 0.05), worse CMTES (rho = −0.68, *p* < 0.01), more severe disability in upper (rho = −0.70, *p* < 0.01) and lower limbs (rho = −0.61, *p* < 0.01) measured by ONLS scores, use of walking aids (*p* < 0.01), and with depression (*p* < 0.01) and fatigue (*p* < 0.01). Worse scores on CMTES (beta = −0.43, *p* < 0.01), BDI (beta = −0.39, *p* < 0.01), and FSS (beta = −0.36, *p* < 0.01) were significant independent predictors of worse QoL in patients with CMT1A (adjusted *R*^2^ = 0.77, *p* < 0.001).

**Conclusion:**

Besides impairment made directly by CMT1A itself, QoL in these patients was also strongly affected by the presence of depression and fatigue. Since CMT1A is still not a curable disease, it is of interest to identify factors associated with QoL that are amenable to treatment.

## Introduction

Charcot-Marie-Tooth (CMT) disease is the most common inherited neuromuscular disease, with a prevalence of up to 82.3 per 1,00,000 inhabitants as registered in Norway (1). An epidemiological study conducted in Serbia reported a prevalence of 9.7/100,000 (2). CMT type 1A (CMT1A) is the most prevalent subtype of the disease, encompassing ~50% of all CMT cases, and it is caused by duplication of the *PMP22* gene (3, 4).

The main clinical characteristics of CMT1A are early-onset, slowly progressive, symmetrical distal muscle weakness, length-dependent sensory impairment, and musculoskeletal deformities, which lead to significant disability and impaired quality of life (QoL). Previous studies reported reduced QoL in patients with CMT (5–15). Most of them emphasized disability, lower limb muscle weakness, gait impairment and use of walking aid (6, 7, 11, 12, 14, 15), or fatigue (9, 12) as the main predictors of reduced QoL in patients with CMT. Socioeconomic and mental aspects of the disease, such as unemployment and emotional distress, were also marked as factors associated with impaired QoL in these patients (5, 11–13, 16). Some studies showed a significant influence of tremor on QoL, especially in pediatric patients with CMT1A (12, 17, 18). Considering the above-mentioned studies, it is not surprising that Redmond et al. (8) found a greater impairment in Physical Functioning, Vitality, and Bodily Pain domains of SF-36 in patients with CMT compared to patients with other chronic and disabling conditions, such as diabetes mellitus, post-stroke, and epilepsy (8).

Most previous studies examined QoL of a heterogeneous group of patients with CMT, including also patients diagnosed solely based on clinical features without a confirmed gene mutation. It is known that the CMT is a heterogeneous disease, presented differently depending on the underlying mutation and disease subtype. Because of the above-mentioned heterogeneity, we included only genetically confirmed patients with CMT1A, which is an important advantage of our study. Furthermore, our study is one of the few that examined the QoL of these patients in developing countries, which is of great importance considering the well-known association of Human Development Index (HDI) and QoL in the general population (19).

We aimed to investigate QoL in Serbian patients with CMT1A and to assess sociodemographic and clinical features associated with their QoL, especially ones that are potentially amenable to treatment or prevention.

## Patients and Method

This study is a part of the Longitudinal Charcot-Marie-Tooth Neuropathy Study of Serbia (LoCh-NeSS) project, which was established to collect data on patients with CMT who have been diagnosed and/or followed at the Neurology Clinic, University Clinical Center of Serbia as the largest tertiary neuromuscular center in the country. Determination of the number of copies of the *PMP22* gene was carried out in the Genetic Laboratory of the Neurology Clinic by the real-time quantification PCR (RT-PCR) using the TaqMan assay on the ABI7500Fast apparatus (Applied Biosystems, USA). The HSA gene was used as an endogenous control. The analysis was carried out in separate reactions, for each sample in triplicate, and the ΔΔCt model was applied in data processing (20). In 10 years, we identified 71 patients with *PMP22* duplication. Among them, 21 patients were lost from follow-up and five refused to be tested. Finally, the total number of subjects included in the study was 45. This study was approved by the Ethical Committee of the Faculty of Medicine, University of Belgrade. Informed consent was obtained from all participants included in the study.

All patients were tested during 2018 and 2019. By reviewing medical records and surveying patients, the following sociodemographic data and clinical features were obtained: gender, age at disease onset, age at the time of referral to the genetic analysis, disease duration, and family history. We differed between juvenile disease onset (if symptoms started before the age of 20) and adult onset of the disease (if the first symptom was noticed after the age of 20). Evaluation of the limb muscle strength was done using the Medical Research Council (MRC) 0–5 point scale (0—no movement, 5—normal strength). Total MRC sum score (MRC-SS) was obtained by adding individual assessments of the following muscle groups on both sides: shoulder abductors, elbow flexors, wrist extensors, hip flexors, knee extensors, and foot dorsiflexors (21). Charcot-Marie-Tooth Examination Score (CMTES) was used to determine the severity of the disease (22). CMTES is a reliable and valid composite scale of seven assessments including symptoms (three items) and signs (four items), and it is a valid measure of overall impairment in patients with CMT. The degree of functional disability was assessed by the Overall Neuropathy Limitation Scale (ONLS) (23). The ONLS score measures the degree to which an individual is able to perform upper and lower limb activities, including turning a key in a lock, washing and brushing hair, doing or undoing buttons or zip, using a knife and fork together, walking, and running. For the evaluation of fatigue, Krupp's Fatigue Severity Scale (FSS) was used (24). It is a 9-item scale that measures the severity of fatigue and its effect on a person's activities and lifestyle. The level of depression was assessed using the Beck Depression Inventory (BDI). Depression was considered if the score was ≥11 (25). Results on the BDI scale in patients with CMT1A were compared with the gender- and age-matched healthy controls. The controls were selected from the large database of neuropsychological and behavioral tests conducted in healthy employees of the Clinic and their relatives. Cognitive functioning was assessed using the Mini-Mental Status Examination (MMSE) scale (26).

Serbian version of the SF-36 questionnaire was used to measure health-related QoL (27). It is a self-report instrument that combines eight general health concepts: physical functioning (PF), physical role (RP), bodily pain (BP), general health (GH), vitality (VT), social functioning (SF), emotional role (RE), and mental health (MH). We also used physical composite score (PCS), mental composite score (MCS), and total SF-36 score to summarize these eight scales. All scores are interpreted on a 0–100 scale, where higher numbers represent better QoL.

The normality of data was evaluated using the Kolmogorov–Smirnov test. For group comparisons, the χ^2^ test, Fisher test, Mann–Whitney *U* test, and Student's *t*-test were used as appropriate. Correlations were estimated using Spearman's rho. To obtain independent predictors of worse QoL, all variables that were associated with total SF-36 score (*p* < 0.01) in univariate analysis were included in the multiple linear regression analysis (stepwise method) with the total SF-36 score being a dependent variable. For all statistical tests, significant testing was two-sided, where *p* < 0.01 was considered highly significant and *p* < 0.05 significant.

## Results

The main sociodemographic and clinical features of Serbian patients with CMT1A are shown in Table 1. Around 50% of patients had disease onset in the first two decades of life, while the median disease duration was 22 years. Mean limb muscle strength measured by the MRC-SS was 49.7 ± 6.4 out of 60. One-third of our patients needed walking aids. We observed a high prevalence of fatigue in patients with CMT1A (56%), whereas almost one-third of our patients had depression in comparison to only 2.2% in healthy controls (*p* < 0.01).

**Table 1:** Main sociodemographic and clinical features of patients with CMT1A (*n* = 45).

**Feature**	**Value**
**Gender (*N*, % of males)**	**18 (40%)**
**Age at testing (years, mean ± SD)**	**50.4 ± 12.6**
**Diagnostic delay (years, median, interquartile range)**	**15 (5–20)**
**Disease duration (years, median, interquartile range)**	**22 (12.5–31.5)**
**Positive family history (*N*, %)**	**38 (84.4%)**
**Disease onset**
** Juvenile (*N*, %)**	**24 (53.3%)**
** Adult (*N*, %)**	**21 (46.7%)**
**Walking aids (*N*, %)**	**15 (33.3%)**
**Scoliosis (*N*, %)**	**19 (42.2%)**
**Tremor (*N*, %)**	**37 (82.2%)**
**Sensory ataxia (*N*, %)**	**20 (44.4%)**
**Perceptive hearing loss (*N*, %)**	**10 (22.2%)**
**Cerebellar dysfunction (*N*, %)**	**8 (17.8%)**
**MMSE below cut-off (*N*, %)**	**4 (8.9%)**
**MRCSS (mean ± SD)**	**49.7 ± 6.4**
**CMTES (mean ± SD)**	**11.3 ± 5.5**
**ONLS score**
** Upper limbs (median, interquartile range)**	**2.0 (1.0–3.0)**
** Lower limbs (median, interquartile range)**	**2.0 (1.75–2.25)**
**BDI above cut-off (*N*, %)**	**13 (28.9%)**
**FSS above cut-off (*N*, %)**	**25 (55.6%)**

*CMT1A, Charcot-Marie-tooth disease type 1; MMSE, Mini Mental State Examination; MRC-SS, Medical Research Council Sum Score; CMTES, Charcot-Marie-Tooth disease Examinations Score; ONLS, Overall Neuropathy Limitation Scale; BDI, Beck Depression Inventory; FSS, Fatigue Severity Scale*.

**Figure 1:**
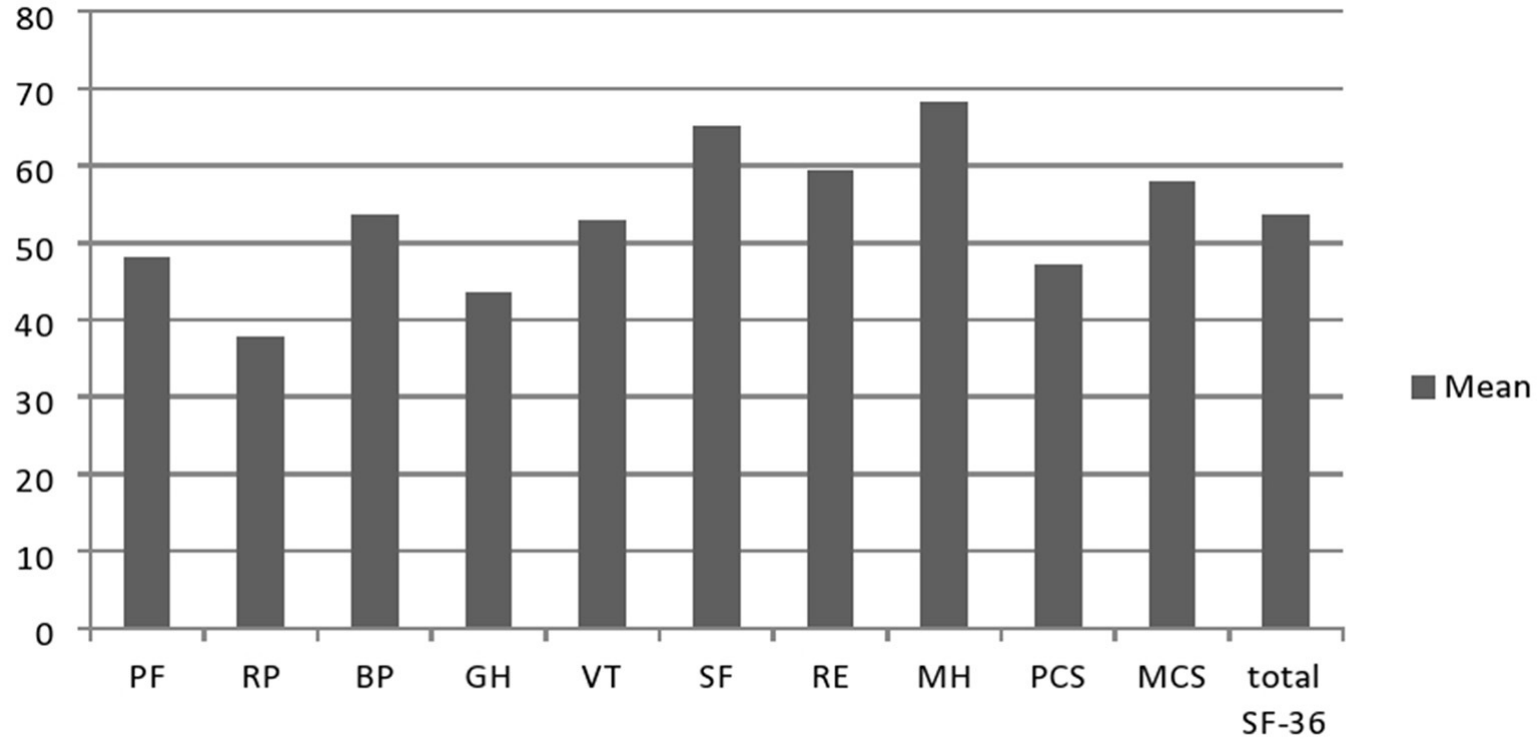
Results on the SF-36 questionnarie in Serbian CMT1A patients (*n* =45). PF, physical functioning; RP, physical role; BP, bodily pain; GH, general health; VT, vitality; SF, social functioning; RE, emotional role; MH, mental health; PCS, physical composite score; MCS, mental composite score.

Results on the SF-36 questionnaire are shown in Figure 1. Furthermore, we analyzed the association of sociodemographic and clinical features with QoL in these patients. Worse scores (PCS, MCS, and total) were associated not only with worse CMTES, MRC-SS, and ONLS for both upper and lower limbs, but also with the presence of fatigue, depression, and walking aid use. Worse PCS and total SF-36 scores, but not MCS, were in association with elder age at testing, longer disease duration, and presence of tremor (Table 2). The following features did not correlate with any of the QoL scales (total SF-36 score, MCS, and PCS): gender, age at onset, sensory ataxia, cerebellar symptomatology, scoliosis, perceptive deafness, and MMSE.

**Table 2:** Association between sociodemographic/clinical features and total SF-36/PCS/MCS scores in patients with CMT1A (*n* = 45).

**Feature**	**Association with total SF-36 score**	**Association with PCS score**	**Association with MCS score**
Age at testing	rho = −0.34*	rho = −0.33*	n.s.
Disease duration	rho = −0.31*	rho = −0.33*	n.s.
Walking aid use (yes vs. no)	36.6 ± 17.1 vs. 62.0 ± 26.3**	24.4 (11.6–37.2) vs. 57.1 (34.8–79.4)**	39.8 (21.5–58.1) vs. 74.0 (51.7–96.3)*
Tremor (yes vs. no)	48.9 (29.4–68.4) vs. 81.7 (53.7–109.7)*	42.0 (0.0–86.2) vs. 78.5 (50.1–106.9)*	n.s.
Fatigue (yes vs. no)	34.9 (20.7–49.1) vs. 77.6 (61.3–93.9)	26.8 (13.2–40.4) vs. 69.5 (51.8–87.1)**	41.3 (25.1–57.4) vs. 80.0 (69.9–90.1)**
Depression (yes vs. no)	22.6 (9.1–36.0) vs. 68.8 (48.4–89.2)**	17.8 (3.8–31.8) vs. 57.1 (33.1–81.0)**	24.3 (13.8–34.8) vs. 75.2 (57.8–92.5)**
MRC-SS	rho = 0.43**	rho = 0.45**	rho = 0.37*
CMTES	rho = −0.68**	rho = −0.70**	rho = −0.56**
ONLS upper limbs	rho = −0.70**	rho = −0.71**	rho = −0.62**
ONLS lower limbs	rho = −0.61**	rho = −0.58**	rho = −0.40**

Results are shown as mean ± SD or as median (interquartile range); SF-36, Short Form 36 Health Survey Questionnaire**;** PCS, Physical Composite Score; MCS, Mental Composite Score; CMT1A, Charcot-Marie-Tooth disease type 1; MMSE, Mini Mental State Examination; MRC-SS, Medical Research Council Sum Score; CMTES, Charcot-Marie-Tooth disease Examinations Score; ONLS, Overall Neuropathy Limitation Scale. * - p <0.05, ** - p <0.01.

In the linear regression analysis, the SF-36 total score was a dependent variable, whereas all parameters that correlated with the total SF-36 score were independent variables. Worse scores on CMTES (beta = −0.43, *p* < 0.01), BDI (beta = −0.39, *p* < 0.01), and FSS (beta = −0.36, *p* < 0.001) were significant independent predictors of worse QoL in patients with CMT1A (adjusted *R*^2^ = 0.77, *p* < 0.001).

## Discussion

Our results showed lower scores in all SF-36 physical domains in Serbian patients with CMT1A compared to the general population of neighborly Croatia (taken as a referent value due to the lack of data for the Serbian general population), which was expected considering CMT1A is an untreatable, progressive, chronic disorder (28). Surprisingly, our patients scored better in two of four mental domains of the SF-36 scale than the general population of Croatia (VT: 52.9 ± 25.14 vs. 51.8 ± 21.5, and MH: 68.3 ± 24.4 vs. 51.8 ± 21.5), which could be explained by the fact that mentioned study was conducted in transitional, post-war Croatia, which could negatively affect these two scores. In addition, it is possible that patients with CMT1A with a long-lasting chronic disease were able to cope with the disease in an adequate way, so their mental domains were higher than generally expected. Compared to Serbian patients with other chronic neuromuscular diseases, the total SF-36 score in patients with CMT1A was like in patients with chronic inflammatory demyelinating polyneuropathy (56.6 ± 25.4) and DM2 (53.6 ± 25.2). On the contrary, the QoL of patients with CMT1A was worse than in patients with MMN (69.8 ± 19.5) and better compared to our patients with myotonic dystrophy type 1 (44.0 ± 21.1) (29–31).

We observed the lowest scores in RP, GH, and PF domains of SF-36. Similar findings with the lowest scores in GH, VT, PF, and RP domains of the SF-36 scale were previously observed in CMT1A (12, 32) and heterogeneous CMT cohorts (6, 14). Otherwise, Roberts-Clarke et al. (14) found similar QoL scores in ten patients with CMT as in the general population, except for GH and PF domains (14). The fact that these authors included only functionally independent patients who were not severely affected by the disease could explain these findings. Accordingly, we observed a greater decrease in PCS than in MCS, which was also reported earlier (5, 9, 12, 14, 32) and well expected as CMT primarily affects limb muscle strength leading to gait disturbances and global deterioration of physical capacity, often with a need for walking aid and loss of independence for daily tasks. On the contrary, Taniguchi et al. (16) showed significant impairment only in emotional and social domains of QoL in Brazilian patients with CMT1A compared to age- and gender-matched control groups and highlighted the importance of including these aspects of QoL in management and future treatment trials of patients with CMT1A (16).

In Serbian patients with CMT1A, decreased QoL in both mental and physical domains (MCS and PCS) was associated with greater muscle weakness, more severe disability, use of walking aids, presence of fatigue, and depression. Three earlier studies showed that disability and limb muscle weakness are the main predictors of worse PCS in a heterogeneous group of patients with CMT (6, 8, 15). Padua et al. (7) performed a study on 89 genetically confirmed patients with CMT1A. Authors reported that the ability to stand independently and the ability to toe- or heel-walk showed the most significant correlation with the SF-36 score (7). Tozza et al. (33) found that more pronounced limb weakness and balance impairment had the greatest self-assessed impact on QoL in patients with CMT1A (33).

Besides physical issues, we noticed a strong correlation between mental aspects of QoL and physical impairment/disability measured through the MRC-SS scale, CMTES scale, and ONLS. Two previous studies also found lower limb weakness/ability to toe-walk as independent predictors of worse scores on mental health domains of SF-36 in patients with CMT1A (7) and CMT as a whole (8). It is already known that lower physical activity levels in physically disabled people correlate with lower scores on mental aspects of QoL (34). This correlation is probably bivariate, because depression or anxiety in physically disabled people leads to even more reduced physical activity and social contacts, and thus to further deterioration of physical abilities (35).

We observed that worse PCS, but not MCS, in patients with CMT1A was associated with elder age, longer disease duration, and presence of tremor. Several studies also observed an association between age/disease duration and PCS, but not MCS (5–7). This is probably due to progressive disease course with an accumulation of neurological deficit, but also due to decline in physical functioning expected with age. Otherwise, elder patients with a longer disease course could be in some way habituated to their disability, which could reflect better scores on the mental domains of QoL. Association between tremor and decreased QoL in patients with CMT1A was already reported in our previous study (12), whereas the most significant influence of tremor on QoL has been shown earlier in the pediatric CMT1A population (17, 18). Considering this, adequate tremor management could be an important tool for improving QoL in these patients.

Besides impairment made by CMT itself (worse score on the CMTES scale), the main predictor of worse QoL in CMT1A was the presence of depression and fatigue. The prevalence of fatigued patients in our CMT1A cohort was 56%, which is in line with previous reports where authors emphasized fatigue as a significant independent predictor of worse QoL in CMT (9, 12, 36).

The prevalence of depression in patients with CMT1A was 28.9 vs. 2.2% in controls. Some previous studies did not find the excess prevalence of depression in patients with CMT (37–40). On the contrary, other authors found a higher prevalence of depression in patients with CMT than in the general population (10, 12, 41). Some of them also highlighted depression as the main predictor of worse MCS (6) or both MCS and PCS (12). Padua, at least partially, explains this discrepancy by the fact that many items of the scales for self-assessment of depression refer to the disease-related symptoms and are, therefore, biased toward depression (38). The discrepancy in depression prevalence and its importance in CMT may be also explained by different scales used in different studies for the assessment of depression, different patients' sample sizes, different subtypes of CMT included, and socioeconomic and cultural differences between countries. Nevertheless, our results suggest that treatment of fatigue and depression could improve QoL in patients with CMT1A.

Our study has several limitations. First, the number of patients is relatively small, but not so small for such a rare disease. However, our study has the advantage that all patients were from the same cultural background. Another limitation of the study is that we did not assess correlations between QoL and nerve conduction study (NCS) results. However, we intentionally decided not to use the NCS part of the CMT Neuropathy Score (CMTNS), but only symptoms and signs scores, so-called CMTES. The reason for this is that previous studies showed that NCS items have a floor effect. For example, the ulnar SAP is absent in almost all patients with CMT, even in mildly affected individuals. Finally, it would be of interest to have prospective, longitudinal data on QoL in CMT1A. Since our patients were tested in the pre-COVID era, we believe that further assessment during the pandemic and in the post-COVID era will be of specific interest based on the previous literature (42). It is our plan to investigate these issues in future studies. While preparing interventional studies in CMT1A, it would be of interest to understand correlations between impairment, disability, QoL, and disease biomarkers (43).

In conclusion, our results confirmed a significant reduction of QoL in patients with CMT1A. We identified several factors associated with QoL, some of them being amenable to treatment, which is of crucial importance in developing new strategies for QoL improvement in untreatable, chronic disorders, such as CMT1A.

## Data Availability Statement

The raw data supporting the conclusions of this article will be made available by the authors, without undue reservation.

## Ethics Statement

The studies involving human participants were reviewed and approved by Ethical Committee of the Faculty of Medicine, University of Belgrade. The patients/participants provided their written informed consent to participate in this study.

## Author Contributions

VI, BB, VS, and AK contributed to the concept or design of the study. VI, BB, AP, MB, IBa, IBo, and AS contributed to the analysis and interpretation of data. VI and BB produced the first draft of the manuscript. All authors provided input into subsequent drafts and reviewed and approved the final version for submission.

## Funding

This study was supported by the Ministry of Education, Science and Technological Development of Serbia, #175083.

## Conflict of Interest

The authors declare that the research was conducted in the absence of any commercial or financial relationships that could be construed as a potential conflict of interest.

## Publisher's Note

All claims expressed in this article are solely those of the authors and do not necessarily represent those of their affiliated organizations, or those of the publisher, the editors and the reviewers. Any product that may be evaluated in this article, or claim that may be made by its manufacturer, is not guaranteed or endorsed by the publisher.
